# A novel prognostic model predicts overall survival in patients with nasopharyngeal carcinoma based on clinical features and blood biomarkers

**DOI:** 10.1002/cam4.3839

**Published:** 2021-05-11

**Authors:** Changchun Lai, Chunning Zhang, Hualiang Lv, Hanqing Huang, Xia Ke, Chuchan Zhou, Hao Chen, Shulin Chen, Lei Zhou

**Affiliations:** ^1^ Department Of Clinical Laboratory Maoming People's Hospital Maoming P. R. China; ^2^ Department Of First Tumor Maoming People's Hospital Maoming P. R. China; ^3^ Department of Pulmonary and Critical Care Medicine Maoming People's Hospital Maoming P. R. China; ^4^ Department of Thoracic Surgery Maoming People's Hospital Maoming P. R. China; ^5^ Department Of Clinical Laboratory Maoming People's Hospital Maoming P. R. China; ^6^ State Key Laboratory of Oncology in South China Collaborative Innovation Center for Cancer Medicine Guangdong Key Laboratory of Nasopharyngeal Carcinoma Diagnosis and Therapy Sun Yat‐sen University Cancer Center Guangzhou P. R. China; ^7^ Research Center for Translational Medicine the First Affiliated Hospital Sun Yat‐sen University Guangzhou P.R. China; ^8^ Department Of Pathology Laboratory Maoming People's Hospital Maoming P. R. China

**Keywords:** lasso regression, model, nasopharyngeal carcinoma, nomogram, prognostic

## Abstract

This study aims to develop and validate a novel prognostic model to estimate overall survival (OS) in nasopharyngeal carcinoma (NPC) patients based on clinical features and blood biomarkers. We assessed the model's incremental value to the TNM staging system, clinical treatment, and Epstein‐Barr virus (EBV) DNA copy number for individual OS estimation. We retrospectively analyzed 519 consecutive patients with NPC. A prognostic model was generated using the Lasso regression model in the training cohort. Then we compared the predictive accuracy of the novel prognostic model with TNM staging, clinical treatment, and EBV DNA copy number using concordance index (C‐index), time‐dependent ROC (tdROC), and decision curve analysis (DCA). Subsequently, we built a nomogram for OS incorporating the prognostic model, TNM staging, and clinical treatment. Finally, we stratified patients into high‐risk and low‐risk groups according to the model risk score, and we analyzed the survival time of these two groups using Kaplan–Meier survival plots. All results were validated in the independent validation cohort. Using the Lasso regression, we established a prognostic model consisting of 13 variables with respect to patient prognosis. The C‐index, tdROC, and DCA showed that the prognostic model had good predictive accuracy and discriminatory power in the training cohort than did TNM staging, clinical treatment, and EBV DNA copy number. Nomogram consisting of the prognostic model, TNM staging, clinical treatment, and EBV DNA copy number showed some superior net benefit. Based on the model risk score, we split the patients into two subgroups: low‐risk (risk score ≤ −1.423) and high‐risk (risk score > −1.423). There were significant differences in OS between the two subgroups of patients. Similar results were observed in the validation cohort. The proposed novel prognostic model based on clinical features and serological markers may represent a promising tool for estimating OS in NPC patients.

## Graphical abstract

1. Currently, the TNM staging system is not adequate for prognosis without considering other clinicopathological factors or serum biomarkers. 2. In this study, we adopt a new algorithm to establish a novel prognostic model based on clinical features and blood biomarkers, which showed better predictive accuracy than traditional TNM staging, clinical treatment, and EBV DNA. 3. The prognostic model represents a promising signature for estimating OS in NPC patients.

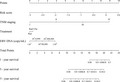

## 1 INTRODUCTION

Nasopharyngeal carcinoma (NPC) is a common malignancy of the head and neck in Southern China and Southeast Asia.^1^ Distant metastasis is a leading cause of treatment failure in patients with NPC; almost 70% of patients are initially diagnosed with locoregionally advanced disease.^2^ Although new radiotherapeutic techniques, chemotherapy regimens, and surgical techniques have improved the survivability of NPC patients, the 5‐year survival rate remains unsatisfactory.^3^

Currently, the tumor–node–metastasis (TNM) staging system is commonly used to determine the prognosis of cancer patients and to guide treatment strategy. However, NPC patients who are at the same TNM stage tend to receive similar treatment, and many patients still show a poor prognosis.^4^ Therefore, TNM staging has some limitations in predicting the survival rate of patients with NPC or in guiding treatments. This because the system is entirely based on the anatomical range of the existing tumors, not on the intrinsic biological heterogeneity of tumors.^5^ Consequently, many biomarkers, such as clinical characteristics,^6^ blood biomarkers,^7^ and radiomics,^8^ have been investigated to improve the prognosis prediction and treatment efficiency of NPC. However, most predictive models are integrated with the TNM staging system to improve the predictive accuracy for clinical outcomes, which makes them inapplicable to patients with uncertain TNM staging. In addition, some models are not widely used in clinical practice, because they are time‐consuming, expensive, carry a high risk of radiation exposure, and are not routine medical examinations in the majority of primary care hospitals.

Recently, more blood biomarkers are used to predict clinical outcomes in many cancers because of their advantages; they are cost‐effective, easily accessible, and straightforward in detecting cancer. Thus, this study aimed to construct a novel prognostic model that predicts the overall survival in NPC patients based on clinical features and routine laboratory blood biomarkers. We assessed the model's incremental value to the TNM staging system, clinical treatment, and Epstein‐Barr virus (EBV) DNA copy number for individual overall survival (OS) estimation. Finally, we validated its effectiveness in patients from the same institution.

## 2 MATERIALS AND METHODS

### 2.1 Patient selection and data collection

Patients with diagnosed NPC from January 2009 to December 2011, who were treated for the first time at Sun Yat‐sen University Cancer Center were retrospectively enrolled. The data were randomly divided into training cohort (2/3) and validation cohort (1/3). This study was performed in accordance with the guidelines outlined in the Declaration of Helsinki and was approved by the Clinical Research Ethics Committee of the Sun Yat‐sen University Cancer Center. All patients provided written informed consent at the first visit to our center. The inclusion criteria for the study were as follows: (i) pathological evidence of NPC, with the absence of any other; (ii) complete baseline clinical information, blood biomarker data, and follow‐up data; (iii) collection of blood biomarker data 1 week before anti‐tumor therapy.

The following clinicopathologic data were collected for each enrolled patient: gender, age, family history of malignant tumors, smoking index (SI): day × the year of cigarette smoking,^9^ body mass index (BMI), TNM staging (assigned according to the 8th AJCC TNM classification),^10^ and clinical treatment. Relevant baseline blood‐biomarkers include white blood cell (WBC), neutrophils (N), lymphocyte (L), monocyte (M), platelet (PLT), hemoglobin (HGB), total protein (TP), albumin (ALB), globulin (GLOB), C‐reactive protein (CRP), apolipoprotein AI (APOA), apolipoprotein B (APOB), dehydrogenase (LDH), high density lipoprotein (HDL), cystatin C (Cys‐C), plasma EBV DNA copy number (EBV DNA), EBV immunoglobulin A/viral capsid antigen (VCA‐IgA), EBV immunoglobulin A/early antigen (EA‐IgA), neutrophil‐to‐lymphocyte ratio (NLR),^11^ derived neutrophil‐lymphocyte ratio (dNLR),^12^ lymphocyte‐to‐monocyte ratio (LMR), platelet‐to‐lymphocyte ratio (PLR), systemic immune‐inflammation index (SII): (platelet × neutrophils)/lymphocyte,^13^ albumin‐to‐globulin ratio (AGR), C‐reactive protein‐to‐albumin ratio (CAR), APOA‐to‐APOB ratio (ABR), advanced lung cancer inflammation index (ALI): (BMI × albumin)/NLR,^14^ prognostic nutritional index (PNI): albumin (g/L) +5 × lymphocyte count × 109/L, and prognostic index (PI): score 0 for CRP 10 mg/L or less and WBC count of 11 × 10^9^/L or less. Patients with only one of these abnormalities were allocated a score of 1; if both of them were elevated, patients were allocated a score of 2.^15^

### 2.2 Patients follow‐up

The follow‐up on patients' survival was performed by referring to the clinic's attendance records, email, and phone calls. All patients were followed‐up after discharge until December 2015. The endpoint of this study was overall survival (OS) was defined as the period from the first time of diagnosing to the last follow‐up or death.

### 2.3 Statistical analyses

Statistical analyses were performed using IBM SPSS Statistical software version 19.0 (IBM Corp.,) and R version 3.6.0 (http://www.R‐project.org). Continuous variables were transformed into categorical variables, and the cut‐off values of all variables were recognized by the R package "survival" and "survminer".^16^ The Pearson Chi‐square test was used to test the differences in distributions of clinical characteristics and blood biomarkers between the training cohort and validation cohort. We used the least absolute shrinkage and selection operator (LASSO) regression to select the most useful prognostic factors in the training cohort. According to the regulation weight λ, LASSO selects variables correlated to the measured outcome by shrinking coefficients’ weights down to zero for the ones not correlated to the OS in NPC patients.^17^ The optimal values of the penalty parameter λ were determined through 10‐fold cross‐validation with the 1‐standard error of the minimum criteria (the 1‐SE criteria).^17^, ^18^ Based on the optimal λ value, we screened a list of prognostic variables with associated coefficients. Then, a novel prognostic model was constructed by calculating the risk score for each patient based on each prognostic variable and its associated coefficient. To compare the predictive accuracy for individual survival between the prognostic model, TNM staging, clinical treatment, and EBV DNA copy number, we evaluated concordance index (C‐index),^19^ time‐dependent ROC (tdROC),^20^ and decision curve analysis (DCA).^21^ Nomograms for the prediction of OS were built (using the rms package in R) based on prognostic model risk score, TNM staging, clinical treatment, and EBV DNA copy number. The calibration plots of nomograms were used to assess the consistency between the predicted survival and the observed survival with bootstrapping (1000 bootstrap resamples).^22^ Finally, the patients in the training and validation cohort were split into low‐risk and high‐risk groups according to the optimal cut‐off value of the prognostic model risk score. Kaplan‐Meier method and log‐rank tests were used to assess differences in OS between the predicted high‐risk and low‐risk groups. Results with two‐sided *p* values of <0.05 were considered statistically significant.

## 3 RESULTS

### 3.1 Baseline clinical characteristics

In the present study, 346 eligible patients were analyzed in the training cohort, and 173 patients were included in the validation cohort. The median follow‐up duration was 51.4 months (interquartile range [IQR]:42.1–67.0 months) for the training cohort and 50.4 months (IQR: 41.9–66.0 months) for the validation cohort. In the training cohort, the 1‐, 3‐, and 5‐year OS rates were 97.4%, 83.8%, and 48.3%, respectively. In the validation cohort, the 1‐, 3‐, and 5‐year OS rates were 94.2%, 84.4%, and 42.8%, respectively.

The optimal cut‐off value for each continuous variable was as follows: age (60 years), smoking index (20.0), BMI (26.33 kg/m^2^), WBC (4.3 × 10^9^/L), neutrophils (7.0 × 10^9^/L), lymphocyte (1.41 × 10^9^/L), monocyte (0.4 × 10^9^/L), platelet (293.0 × 10^9^/L), hemoglobin (130.0 g/L), neutrophil‐to‐lymphocyte ratio (3.91), derived neutrophil‐to‐lymphocyte ratio (2.46), lymphocyte‐to‐monocyte ratio (3.4), platelet‐to‐lymphocyte ratio (208.89), systemic immune‐inflammation index (1141.96), total protein (77.2 g/L), albumin (42.4 g/L), globulin (33.1 g/L), albumin‐to‐globulin ratio (1.36), CRP (5.47 mg/L), CRP‐to‐albumin ratio (0.16), apo A (1.28 g/L), apo B (1.03 g/L), apo A–to–apo B ratio (0.96), LDH (167.5 U/L), HDL (1.16 U/L), cystatin C (0.94 mg/L), advanced lung cancer inflammation index (262.33), and prognostic nutritional index (47.35). Patients’ clinical characteristics and blood biomarkers for the patients are listed in Table 1. There was no significant difference in the distribution of clinical characteristics and blood‐biomarkers between training cohort and validation cohort.

**TABLE 1:** Demographics and clinical characteristics of patients in the training and validation cohort

Characteristic	Training cohort	Validation cohort	χ^2^ value	*p* value
	n = (346)	n = (173)		
	No. (%)	No. (%)		
Gender				
Male	264 (76.3%)	121 (69.9%)	2.435	0.119
Female	82 (23.7%)	52 (30.1%)		
Age (years)				
≤60	310 (89.6%)	150 (86.7%)	0.956	0.328
>60	36 (10.4%)	23 (13.3%)		
Family history				
Yes	90 (26.0%)	47 (27.2%)	0.079	0.778
No	256 (74.0%)	126 (72.8%)		
Smoking index^a^				
≤20.0	226 (65.3%)	103 (59.5%)	1.661	0.198
>20.0	120 (34.7%)	70 (40.5%)		
BMI (kg/m^2^)				
≤26.33	298 (86.1%)	155 (89.6%)	1.250	0.264
>26.33	48 (13.9%)	18 (10.4%)		
TNM stage^b^				
I	12 (3.5%)	5 (2.9%)	1.965	0.580
II	45 (13.0%)	24 (13.9%)		
III	172 (49.7%)	76 (43.9%)		
IV	117 (33.8%)	68 (39.3%)		
Treatment				
Rad	58 (16.8%)	32 (18.5%)	0.242	0.623
Rad and Che	288 (83.2%)	141 (81.5%)		
WBC (10^9^/L)				
≤4.3	57 (16.5%)	29 (16.8%)	0.007	0.933
>4.3	289 (83.5%)	144 (83.2%)		
Neutrophils (10^9^/L)				
≤7.0	306 (88.4%)	148 (85.5%)	0.879	0.348
>7.0	40 (11.6%)	25 (14.5%)		
Lymphocyte (10^9^/L)				
≤1.41	145 (41.9%)	75 (43.4%)	0.099	0.753
>1.41	201 (58.1%)	98 (56.6%)		
Monocyte (10^9^/L)				
≤0.4	175 (50.6%)	82 (47.4%)	0.466	0.495
>0.4	171 (49.4%)	91 (52.6%)		
Platelet (10^9^/L)				
≤293.0	298 (86.1%)	154 (89.0%)	0.857	0.355
>293.0	48 (13.9%)	19 (11.0%)		
HGB (g/L)				
≤130.0	106 (30.6%)	61 (35.3%)	1.130	0.288
>130.0	240 (69.4%)	112 (64.7%)		
NLR				
≤3.91	263 (76.0%)	126 (72.8%)	0.621	0.431
>3.91	83 (24.0%)	47 (27.2%)		
Dnlr				
≤2.46	254 (73.4%)	121 (69.9%)	0.692	0.405
>2.46	92 (26.6%)	52 (30.1%)		
LMR				
≤3.4	141 (40.8%)	76 (43.9%)	0.479	0.489
>3.4	205 (59.2%)	97 (56.1%)		
PLR				
≤208.89	277 (80.1%)	140 (80.9%)	0.055	0.815
>208.89	69 (19.9%)	33 (19.1%)		
SII				
≤1141.96	294 (85.0%)	144 (83.2%)	0.263	0.608
>1141.96	52 (15.0%)	29 (16.8%)		
TP (g/L)				
≤77.2	273 (78.9%)	128 (74.0%)	1.585	0.208
>77.2	73 (1.1%)	45 (26.0%)		
ALB (g/L)				
≤42.4	132 (38.2%)	63 (36.4%)	0.148	0.701
>42.4	214 (61.8%)	110 (63.6%)		
GLOB (g/L)				
≤33.1	274 (79.2%)	139 (80.3%)	0.095	0.758
>33.1	72 (20.8%)	34 (19.7%)		
AGR				
≤1.36	108 (30.6%)	45 (26.0%)	1.406	0.236
>1.36	240 (69.4%)	128 (74.0%)		
CRP (mg/L)				
≤5.47	268 (77.5%)	132 (76.3%)	0.087	0.768
>5.47	78 (22.5%)	41 (23.7%)		
CAR				
≤0.16	282 (81.56%)	139 (80.3%)	0.101	0.751
>0.16	64 (18.5%)	34 (19.7%)		
APOA (g/L)				
≤1.28	167 (48.3%)	81 (46.8%)	0.097	0.756
>1.28	179 (51.7%)	92 (53.2%)		
APOB (g/L)				
≤1.03	218 (63.0%)	105 (60.7%)	0.262	0.609
>1.03	128 (37.0%)	68 (39.3%)		
ABR				
≤0.96	40 (11.6%)	19 (11.0%)	0.038	0.845
>0.96	306 (88.4%)	154 (89.0%)		
LDH (U/L)				
≤167.5	193 (55.8%)	96 (55.5%)	0.004	0.950
>167.5	153 (44.2%)	77 (44.5%)		
HDL (U/L)				
≤1.16	179 (51.7%)	81 (46.8%)	1.114	0.291
>1.16	167 (48.3%)	92 (53.2%)		
Cys‐C (mg/L)				
≤0.94	222 (64.2%)	101 (58.4%)	1.640	0.200
>0.94	124 (35.8%)	72 (41.6%)		
EBV DNA, copy/mL				
<10^3^	169 (48.8%)	70 (40.5%)	4.369	0.358
10^3^–9,999	72 (20.8%)	36 (20.8%)		
10^4^–99,999	58 (16.8%)	39 (22.5%)		
10^5^–999,999	29 (8.4%)	17 (9.8%)		
≥10^6^	18 (5.2%)	11 (6.4%)		
VCA‐IgA				
<1:80	59 (17.1%)	28 (16.2%)	0.081	0.960
1:80–1:320	208 (60.1%)	106 (61.3%)		
≥1:640	79 (22.8%)	39 (22.5%)		
EA‐IgA				
<1:10	116 (32.7%)	49 (28.3%)	1.338	0.512
1:10–1:20	110 (31.8%)	60 (34.7%)		
≥1:40	123 (35.5%)	64 (37.0%)		
ALI				
≤262.33	94 (27.2%)	50 (28.9%)	0.173	0.677
>262.33	252 (72.8%)	123 (71.1%)		
PNI				
≤47.35	63 (18.2%)	33 (19.1%)	0.058	0.810
>47.35	283 (81.8%)	140 (80.9%)		
PI				
0	275 (79.5%)	141 (81.5%)	0.644	0.725
1	64 (18.5%)	30 (17.3%)		
2	7 (2.0%)	2 (1.2%)		

Abbreviations: BMI, body mass index; TNM, Tumor Node Metastasis stage; Rad, radiotherapy; Che, chemotherapy; WBC, white blood cell; HGB, hemoglobin; NLR, neutrophil/lymphocyte ratio; dNLR, neutrophil/WBC‐neutrophil ratio; LMR, lymphocyte/monocyte ratio; PLR, platelet/lymphocyte ratio; SII, systemic immune‐inflammation index; TP, total protein; ALB, albumin; GLOB, globulin; AGR, ALB/GLOB ratio; CRP, C‐reactive protein; CAR, C‐reactive protein/albumin ratio; APOA, apolipoprotein AI; APOB, apolipoprotein B; ABR, APOA/APOB ratio; LDH, lactic dehydrogenase; HDL, high density lipoprotein; Cys‐C, cystatin C; EBV, Epstein‐Barr virus; VCA‐IgA, EBV immunoglobulin A/viral capsid antigen; EA‐IgA, EBV immunoglobulin A/early antigen; ALI, advanced lung cancer inflammation index; PNI, prognostic nutritional index; PI, prognostic index.

^a^ Smoking index: the number of cigarettes smoked each day × the year of cigarette smoking

^b^ TNM stage was classified according to the AJCC 8th TNM staging system

### 3.2 Construction of the novel prognostic model

**FIGURE 1:**
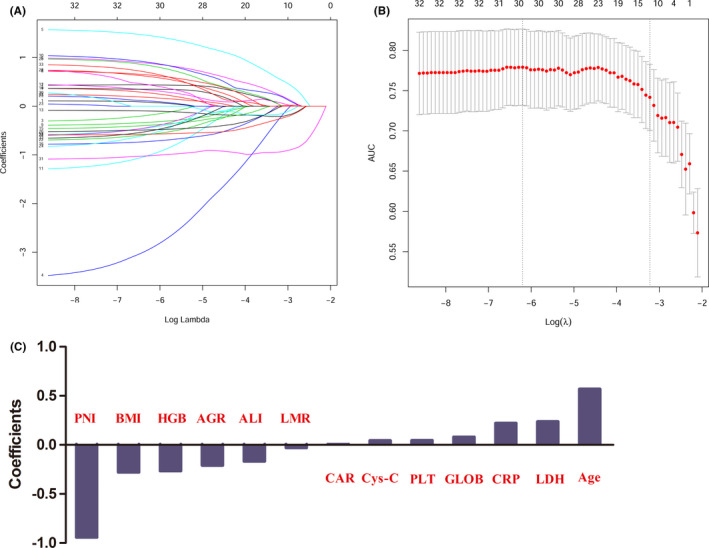
Potential predictors' selection using LASSO regression model

To find the prognostic variables in the training cohort, we used a LASSO regression analysis model. Figure 1A shows the change in the trajectory of each prognostic variable. Moreover, we plotted the partial likelihood deviance versus log (λ) in Figure 1B, where λ was the tuning parameter. The value of λ was 0.03987 and was chosen by 10‐fold cross‐validation via the 1‐SE criteria. So, we obtained 13 variables with nonzero coefficients at the value λ chosen by the cross‐validation. These prognostic variables included age, BMI, hemoglobin (HGB), platelet (PLT), lymphocyte‐to‐monocyte ratio (LMR), CRP, CRP‐to‐albumin ratio (CAR), globulin (GLOB), albumin‐to‐globulin ratio (AGR), LDH, cystatin C (Cys‐C), advanced lung cancer inflammation index (ALI), and prognostic nutritional index (PNI). The coefficients of each prognostic variable are presented in Figure 1C. Then the prognostic model risk score for each patient was computed according to the summation of 13 variables multiplied by a coefficient generated from the LASSO regression: The prognostic model risk score = −0.680 + (0.569 × age) − (0.280 × BMI + (0.101 × HGB) − (0.554 × PLT) + (0.197 × LMR) − (0.199 × CRP) + (0.186 × CAR) + (1.248 × GLOB) − (0.137 × AGR) − (0.194 × LDH) + (1.248 × Cys‐C) − (0.137 × ALI) − (0.194 × PNI). Each variable was valued as 0 or 1; a value of 0 was assigned when the variable was less than or equal to the corresponding cut‐off value, and a value of 1 otherwise.

### 3.3 Predictive accuracy of the novel prognostic model, compared with TNM staging, clinical treatment, and EBV DNA copy number

**FIGURE 2:**
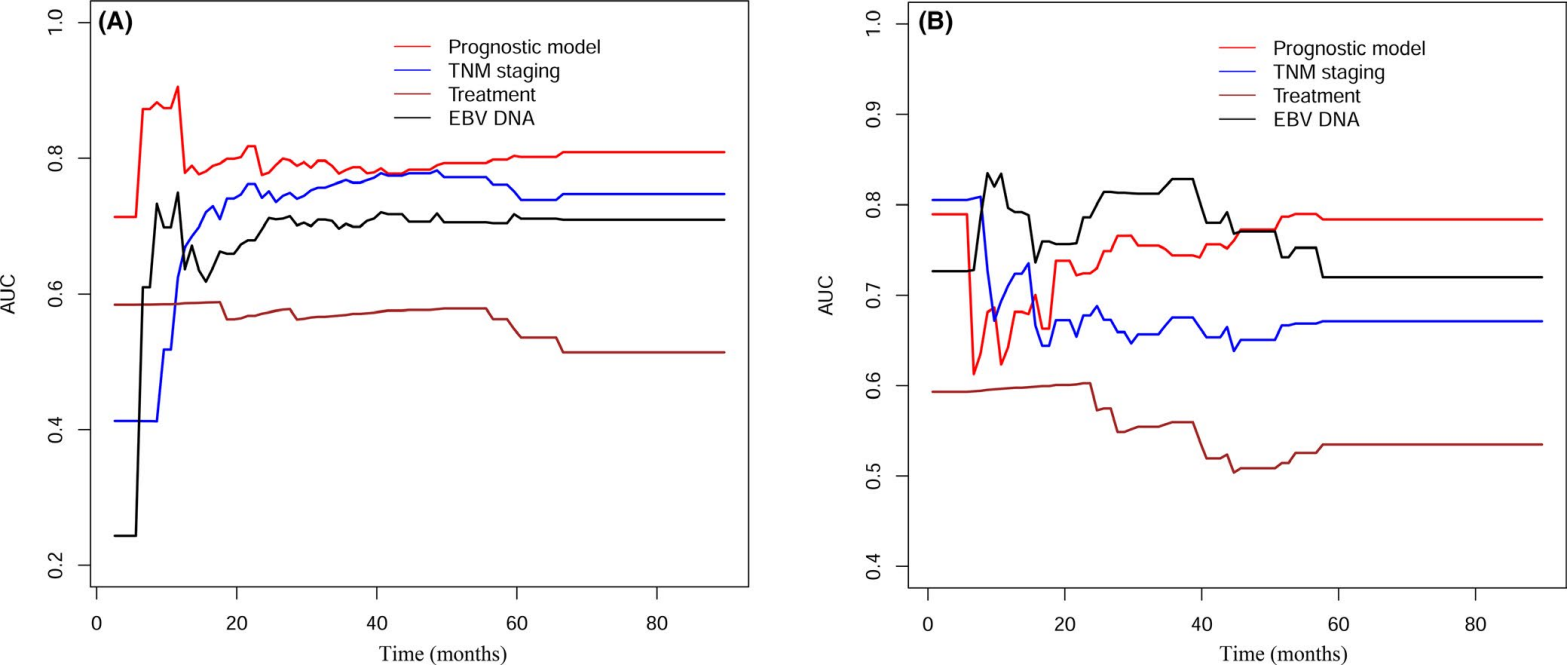
Comparison of predictive accuracy between prognostic model, TNM staging, and clinical treatment using time‐dependent ROC curves in training cohort and validation cohort

**FIGURE 3:**
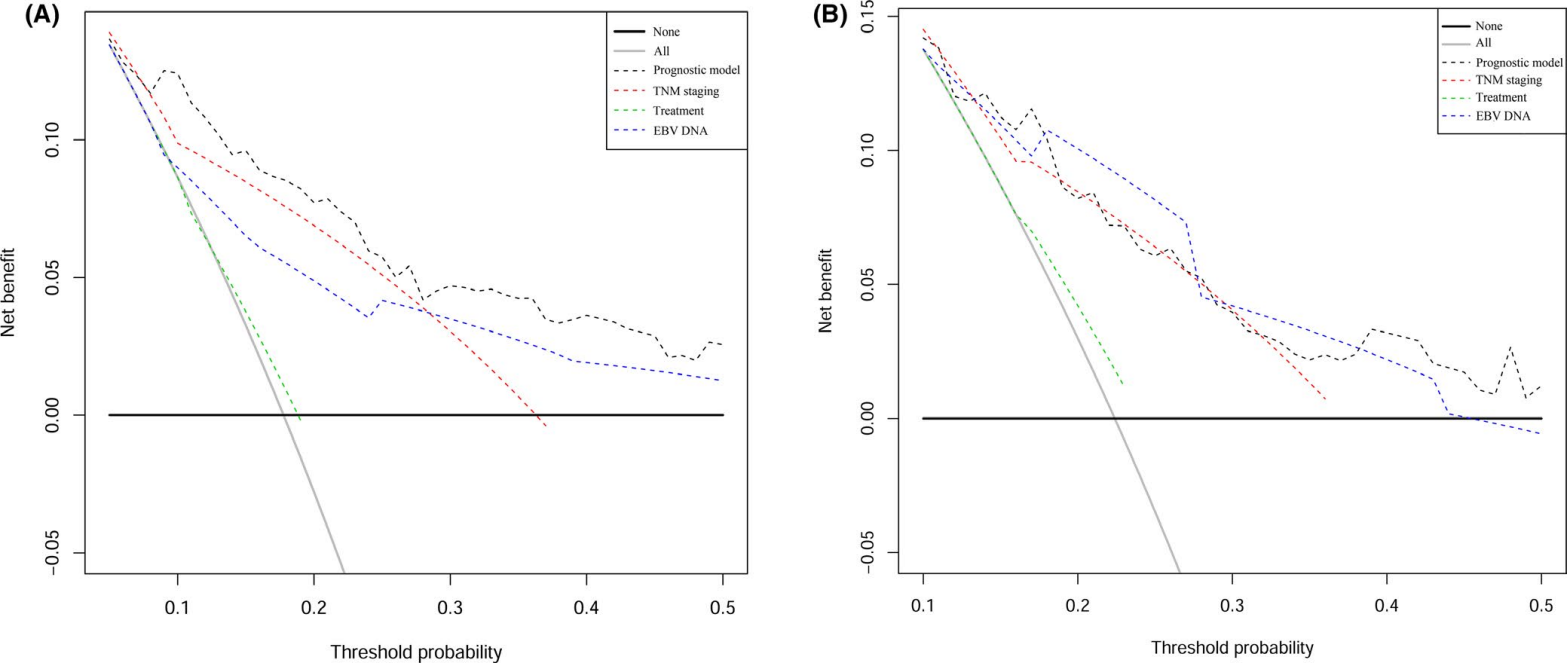
Decision curve analysis for each model in training cohort and validation cohort

As shown in Table 2, in the training cohort, the C‐index of the prognostic model was 0.786 (95% confidence interval [CI]: 0.728–0.844), which was higher than the C‐indices of the TNM staging (0.740, 95% CI: 0.690–0.790), clinical treatment (0.554, 95% CI: 0.521–0.586), and EBV DNA copy number (0.691, 95% CI: 0.623–0.758). The C‐index of the prognostic model was significantly higher than the C‐index of the clinical treatment (*p* < 0.001), and that of EBV DNA copy number (*p* = 0.013). In the validation cohort, the C‐index of the prognostic model was higher than that of TNM staging and clinical treatment, but was a little lower than that of EBV DNA copy number. Subsequently, we compared the area under the ROC curve (AUC) between the novel prognostic model, TNM staging, clinical treatment, and EBV DNA copy number using tdROC. In general, the AUC of our novel prognostic model was higher than the others, both in the training cohort (Figure 2A) and the validation cohort (Figure 2B). Finally, the DCA showed that the prognostic model had a better overall net benefit than that of TNM staging, clinical treatment, and EBV DNA copy number across a wide range of reasonable threshold probabilities in the training cohort (Figure 3A) and the validation cohort (Figure 3B). These results indicated that the novel prognostic model displayed better accuracy in predicting OS compared with TNM staging, clinical treatment, and EBV DNA copy number.

**TABLE 2:** The C‐index of the prognostic model, TNM staging, Treatment, and EBV DNA for prediction of OS in the training cohort and validation cohort

Factors	C‐index (95% CI)	*p*
For training cohort		
Prognostic model	0.786 (0.728 ~ 0.844)	
TNM staging	0.740 (0.690 ~ 0.790)	
Treatment	0.554 (0.521 ~ 0.586)	
EBV DNA	0.691 (0.623 ~ 0.758)	
Prognostic model versus TNM staging		0.067
Prognostic model versus Treatment		<0.001
Prognostic model versus EBV DNA		0.013
For validation cohort		
Prognostic model	0.697 (0.612 ~ 0.734)	
TNM staging	0.655 (0.575 ~ 0.734)	
Treatment	0.529 (0.470 ~ 0.588)	
EBV DNA	0.734 (0.659 ~ 0.813)	
Prognostic model versus TNM staging		0.310
Prognostic model versus Treatment		<0.001
Prognostic model versus EBV DNA		0.511

C‐index, concordance index; CI, confidence interval; P values are calculated based on normal approximation using function rcorrp.cens in Hmisc package.

### 3.4 Building and validating a predictive nomogram

**FIGURE 4:**
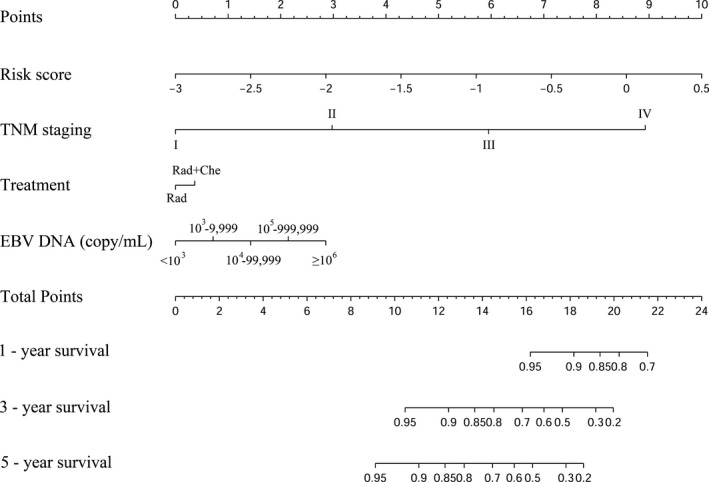
The nomogram was used to estimate OS for NPC patients in the training cohor

**FIGURE 5:**
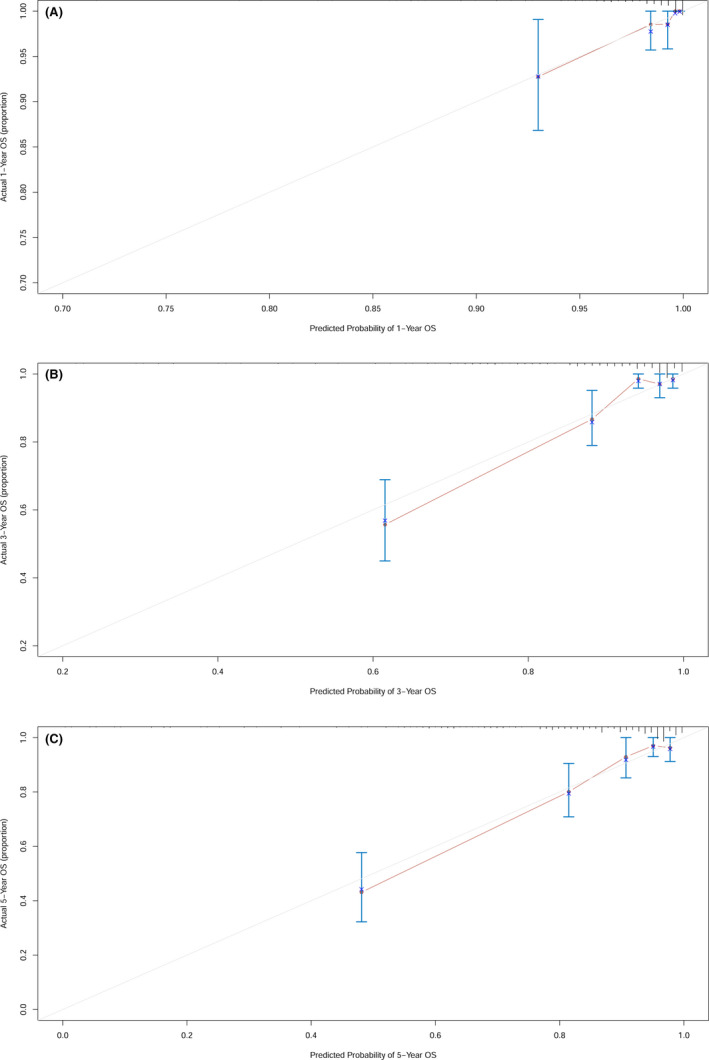
The calibration plot for the nomograms at 1‐, 3‐, 5‐ year in the training cohort

The prognostic model risk score, TNM staging, clinical treatment, and EBV DNA copy number were integrated into nomograms to predict the 1‐, 3‐, and 5‐year OS in the training cohort (Figure 4). Each variable was assigned a corresponding point value based on its contribution to the model. The point values for all the predictor variables are summed to arrive at the "total points" axis, and then a line is drawn vertically down from total points to predict the patient's probability of OS at 1‐, 3‐, and 5‐year. Finally, a calibration plot was used to visualize the performance of the nomogram. The nomogram‐predicted outcomes for 1‐, 3‐, and 5‐year OS were plotted on the x‐axis, while the actual observed outcome on the y‐axis. The 45° line represented the best prediction, the solid dark red line represented the performance of the nomograms. The calibration curve showed that the 1‐, 3‐, and 5‐year OS predicted by the nomograms were consistent with actual observations (Figure 5), indicating that the nomograms performed well. The nomograms and calibration curves in the validation cohort are shown in Figure S1 and Figure S2, respectively.

### 3.5 Survival analyses of NPC patients according to prognostic model risk score

**FIGURE 6:**
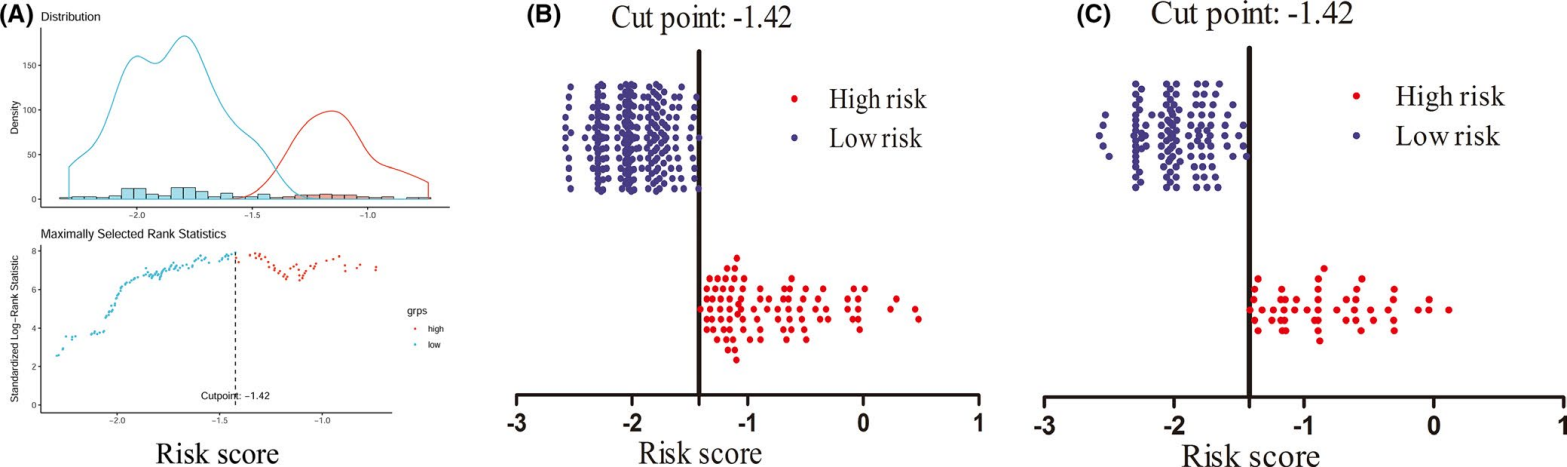
The optimal cut‐off value of prognostic model risk score using R package "survival," and the distribution of the prognostic model risk score in the training cohort and validation cohort

The optimal cut‐off value of the prognostic model risk score for predicting survival was determined to be −1.423 by R package “survminer” (Figure 6A). We classified patients into two different subgroups based on the cut‐off value: low‐risk group (risk score ≤ −1.423), and high‐risk group (risk score > −1.423). The distribution of the prognostic model risk score in the training and the validation cohort are shown in Figure 6B and Figure 6C, respectively.

**FIGURE 7:**
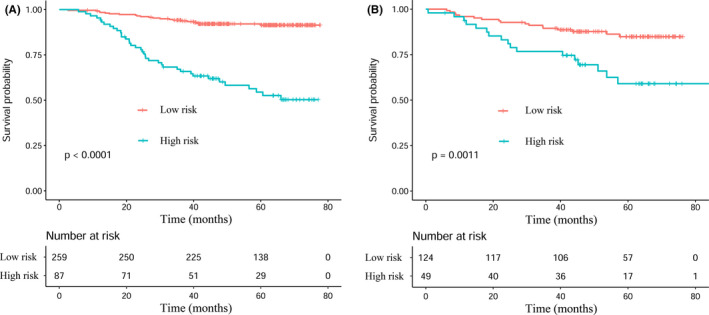
Kaplan–Meier analyses of OS according to the prognostic model risk score classifier in subgroups of NPC patients in the training cohort and the validation cohort

In the training cohort, for the high‐risk group, the median OS was 44.4 months (IQR: 24.7–66.1). The probabilities of OS at 1‐, 3‐, and 5‐year were 95.4%, 63.2%, and 33.3%, respectively. For the low‐risk group, the median OS was 61.2 months (IQR: 44.6–67.8). The probabilities of OS at 1‐, 3‐, and 5‐year were 98.1%, 90.7%, and 53.3%, respectively. In the validation cohort, the low‐risk group showed higher survival probabilities than did the high‐risk group at 1‐, 3‐, and 5‐year (Table 3). Kaplan–Meier curves were compared to assess the differences in survival between low‐risk and high‐risk groups. The low‐risk group showed significantly longer OS than the high‐risk group for both cohorts (*p* < 0.05; Figure 7).

**TABLE 3:** OS and OS rate in high‐risk and low‐risk groups according to the model risk score in the training and validation cohort.

Parameter	Training cohort			Validation cohort		
	High‐Risk Group	Low‐Risk Group	Total	High‐Risk Group	Low ‐Risk Group	Total
No. of patients	87	259	346	49	124	173
Median (IQR)	44.4 (24.7–66.1)	61.2 (44.6–67.8)	51.4 (42.1–67.0)	45.8 (26.1–64.1)	53.5 (43.0–66.3)	50.4 (41.9–66.0)
No. of OS						
1‐Year	83 (95.4%)	254 (98.1%)	337 (97.4%)	44 (89.8%)	119 (96.0%)	163 (94.2%)
3‐Year	55 (63.2%)	235 (90.7%)	290 (83.8%)	36 (73.5%)	110 (88.7%)	146 (84.4%)
5‐Year	29 (33.3%)	138 (53.3%)	167 (48.3%)	17 (34.7%)	57 (46.0%)	74 (42.8%)

Abbreviations: OS, overall survival; IQR, interquartile range.

## 4 DISCUSSION

In this study, we successfully established a novel prognostic model based on clinical features and blood biomarkers of NPC for individualized prediction of the OS. The novel prognostic model showed better predictive accuracy and discrimination compared with the traditional AJCC TNM staging system, clinical treatment, and EBV DNA copy number. The model successfully splits NPC patients into high‐risk and low‐risk groups, and both groups exhibited significant differences in OS.

The present prognostic model consisted of 13 prognostic variables: age, BMI, HGB, PLT, LMR, CRP, CAR, GLOB, AGR, LDH, Cys‐C, ALI, and PNI. All the prognosis variables were associated with survival in NPC patients except ALI.^23^, ^24^, ^25^, ^26^, ^27^, ^28^, ^29^ These were credible evidence supporting our analysis results. The ALI was devised to assess the degree of systemic inflammation in patients with advanced non‐small‐cell lung cancer patients.^30^ Subsequently, this index was found to be a prognostic factor of survival in some cancers.^31^, ^32^, ^33^ The difference between the ALI and other inflammatory markers was that the former contained not only indices related to inflammation but also BMI, which was reported to correlate with the sarcopenic status.^32^ So, this was the first study to indicate ALI as a prognostic marker in NPC patients.

Subsequently, we compared the predictive accuracy and discrimination of the novel prognostic model with TNM staging, clinical treatment, and EBV DNA copy number using C‐index, tdROC, and DCA. We found that the prognostic model had good predictive accuracy and discriminatory power than TNM staging, clinical treatment, and EBV DNA copy number in the training cohort. Similar results were observed in the validation cohort except for the EBV DNA copy number. The C‐index of the prognostic model was slightly lower than that of the EBV DNA copy number, but they were not significantly different. The most likely explanation was that this was a retrospective analysis, and there may have been some potential patient selection bias. Then the nomogram consisting of the prognostic model, TNM staging, clinical treatment, and EBV DNA copy number showed superior net benefit. Finally, according to the model's risk score, we split the patients into two subgroups: low‐risk and high‐risk, There were significant differences in OS between the two subgroups of patients. These results indicated that the novel prognostic model had good predictive accuracy and discrimination for estimating OS in NPC patients.

Although previous studies had established some models for predicting NPC survival, this study still has several merits compared with other studies. First, the prognostic model only included basic clinical and routine laboratory data, which did not include markers that are not routinely available, such as EBV DNA,^34^ and circulating tumor cells (CTC).^35^, ^36^ This model was low‐cost, non‐invasive, convenient, and has no risk of radiation exposure. So, this model could be widely and safely used in clinical practice, especially in primary hospitals. Second, the prognostic model was constructed using a new algorithm: LASSO regression analysis, as a statistical method for screening variables to establish the prognostic model. The algorithm enabled adjusting for the model's overfitting, thus avoiding extreme predictions. Therefore, predictive accuracy could be improved significantly. This approach had been applied in a few studies.^37^, ^38^, ^39^, ^40^ Third, many previous models integrated TNM staging and/or clinical treatment to improve predictive accuracy for clinical outcomes,^26^, ^41^, ^42^, ^43^, ^44^, ^45^, ^46^ which made them inapplicable to patients who have uncertain TNM staging. Our model can be used for those patients because it does not include TNM staging. Fourth, although another group, Sun et al.,^40^ had established two nomograms to predict the benefit of concurrent chemotherapy in stage II‐IVa NPC patients, their research did not analyze other important biomarkers in the blood (except for EBV DNA). Additionally, for OS, the C‐indices of the nomograms only ranged from 0.700 to 0.711. In our study, we established a novel prognostic model based on the clinical features and blood biomarkers (including inflammation‐based scoring systems, liver function markers, and others). The C‐index of our model was 0.786. Clinicians could benefit from combining our model with others.

There were also several drawbacks to this study. This was a retrospective analysis, so selection bias might have occurred, and it was inevitable that there will be some patients on censoring and lost to follow‐up. The treatment effect heterogeneity for metachronous metastatic cancer might have confounding effects. The endpoint of this study was OS, and we did not assess the model's suitability to predict disease‐free survival (DFS), distant metastasis‐free survival (DMFS), and locoregional relapse‐free survival (LRFS) in NPC patients.^47^ It may be better if the endpoint combined OS with DFS and DMFS. Furthermore, because other medical institutions may lack the facilities to detect some indicators (such as Cys‐C, CRP, and LDH), this may limit the wide application of the model in other centers. This retrospective study was performed in EBV‐related NPC patients, it is unknown whether it can be used for non‐EBV‐related NPC, and this would be needed to be confirmed in non‐EBV‐related NPC patients. Finally, our study was a single‐institutional study with a relatively small sample size. Thus, a large‐scale and multicenter validation of the model will be needed in the future.

In conclusion, we have established a novel prognostic model based on clinical features and blood biomarkers, which showed better predictive accuracy than traditional TNM staging, clinical treatment, and EBV DNA copy number alone. The nomograms comprising the prognostic model, TNM staging, clinical treatment, and EBV DNA can reinforce the capability of the prognostic model. Therefore, our convenient, low‐cost, non‐invasive, no risk of radiation exposure, and straightforward prognostic model may useful for clinicians in making decisions, counseling individual patients, and scheduling follow‐ups for NPC patients.

## CONFLICT OF INTEREST

The authors declare that they have no conflict of interest.

## Supporting information

Fig S1

Fig S2

## Data Availability

The data sets analyzed during the current study are not publicly available due to patient privacy concerns, but are available from the corresponding author on reasonable request.
