# Supplementary figures and images for: A novel prognostic model predicts overall survival in patients with nasopharyngeal carcinoma based on clinical features and blood biomarkers
Source: Cancer Med. 2021 May 11;10(11):3511–23. doi: 10.1002/cam4.3839 (PMC8178501; doi:10.1002/cam4.3839)

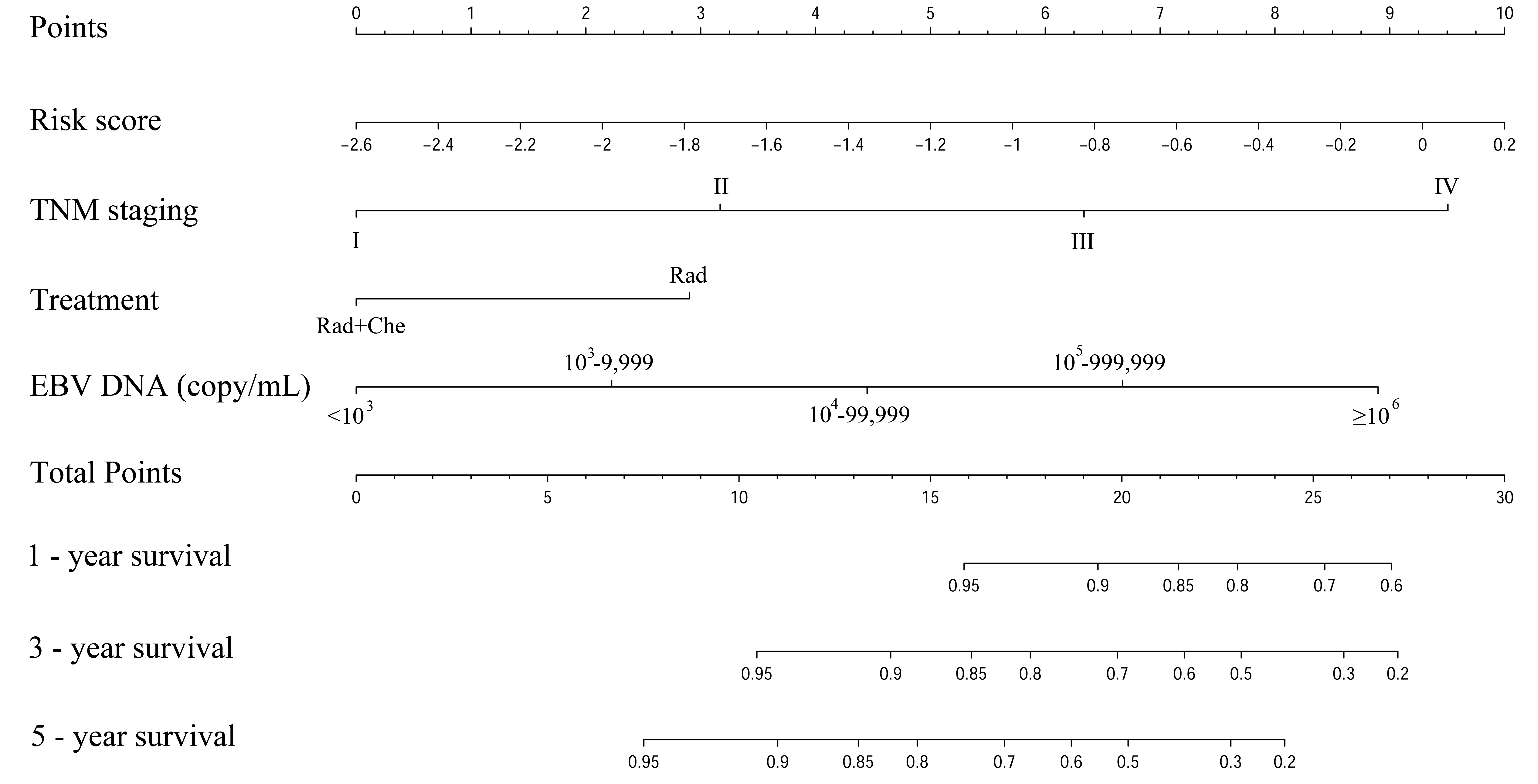

Supplement: Supplementary file 1 — Fig S1 [file CAM4-10-3511-s002.tif]

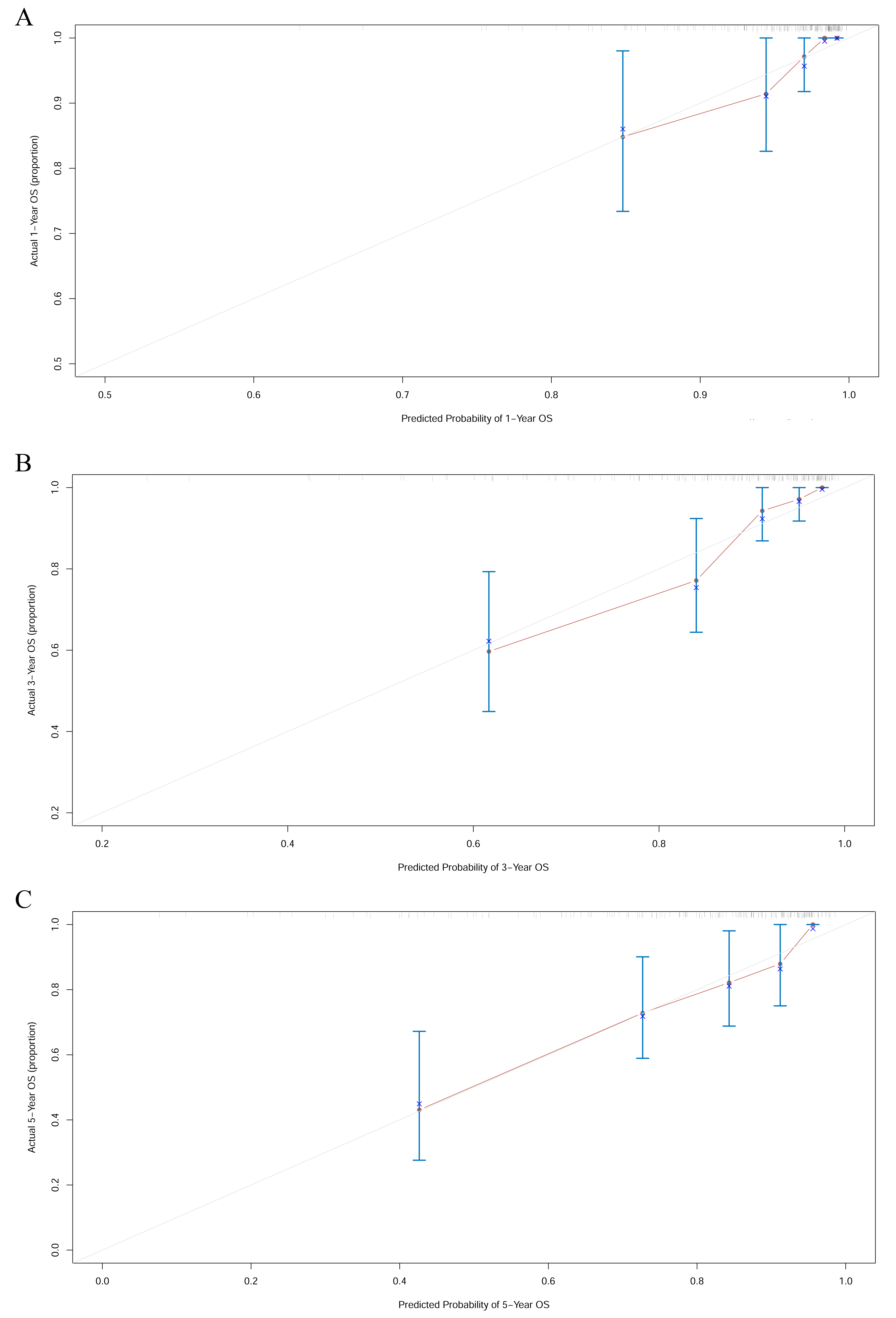

Supplement: Supplementary file 2 — Fig S2 [file CAM4-10-3511-s001.tif]
